# Epithelial autophagy controls chronic colitis by reducing TNF-induced apoptosis

**DOI:** 10.1080/15548627.2018.1450021

**Published:** 2018-05-25

**Authors:** Johanna Pott, Kevin J. Maloy

**Affiliations:** Sir William Dunn School of Pathology, University of Oxford, Oxford, UK

**Keywords:** Apoptosis, autophagy, intestinal epithelial cell, colitis, Crohn's disease, ulcerative colitis

## Abstract

Genome-wide association studies (GWAS) linking polymorphisms in *ATG16L1* with susceptibility to inflammatory bowel disease (IBD) have prompted mucosal immunologists to investigate the functional roles of macroautophagy/autophagy in different cell types in the gut. Here we present a recent study that addressed 2 key questions: in which cell type is autophagy deficiency most detrimental during chronic colitis and what is the functional role of autophagy in those cells? We report that autophagy in intestinal epithelial cells (IECs) acts to limit intestinal inflammation by protecting them from TNF-induced apoptosis and we discuss the potential implications for IBD treatment.

Inflammatory bowel disease is a complex inflammatory disorder of the gastrointestinal tract thought to arise from a dysregulated immune response towards the microbiota. Although many pathogenic networks underlying disease development have been identified, the exact etiology is unknown, reflecting the complexity and diversity of the disease manifestations. Polymorphisms in several autophagy-related genes, such as *ATG16L1* and *IRGM*, have been linked to IBD by GWAS. Autophagy is a versatile cellular degradation pathway implicated in many stress response mechanisms fundamentally important in nearly every cell type. Therefore, the genetic links identified by GWAS do not give any mechanistic information about the functional consequences, or about the cell types in which the autophagy pathway is important during chronic intestinal inflammation.

Recently, we addressed this issue by utilizing a Cre-lox genetic approach to generate transgenic mouse lines in which the IBD-associated autophagy gene *Atg16l1* was selectively ablated in distinct cellular compartments. To assess the impact on chronic intestinal inflammation, we used an IBD model triggered by infection with the opportunistic bacterial pathogen *Helicobacter hepaticus* and simultaneous inhibition of the regulatory arm of the immune response with anti-IL10R blocking antibody. Although previous studies in other systems suggest that autophagy can limit release of pro-inflammatory cytokines by myeloid cells (macrophages and dendritic cells), we found that selective ablation of *Atg16l1* in the myeloid compartments has little impact on this bacterially triggered chronic intestinal inflammation. In contrast, mice with selective ablation of autophagy in IECs (*Atg16l1*^VC^ mice) exhibit severely exacerbated intestinal pathology, characterized by increased accumulation of CD4^+^ T cells in the lamina propria and elevated levels of pro-inflammatory cytokines.

The increased pathology in *Atg16l1*^VC^ mice is also accompanied by a higher number of apoptotic cells in the epithelium. Increased epithelial cell death could result in impaired barrier function leading to exacerbated pathology; however, it could also be just a consequence of the elevated inflammatory cytokines present in *Atg16l1*^VC^ mice. This led us to investigate whether autophagy intrinsically regulates cytokine-induced apoptosis in IECs. Using complementary approaches, we found that autophagy directly controls apoptosis in epithelial cells. First, utilizing the recently developed ‘organoid’ system to culture primary IECs ex vivo, we found that *Atg16l1-*deficient IECs show increased induction of apoptosis following exposure to pro-inflammatory cytokines (TNF + IFNG/IFNγ) compared to wild-type IECs. Second, in vivo injection of TNF induces higher numbers of apoptotic IECs in the small intestine of *Atg16l1*^VC^ mice compared to wild-type mice. Of note, increased apoptosis in autophagy-deficient IECs is only observed following inflammatory cytokine stimulation or in the context of chronic inflammation; at steady-state, IECs cope well with the lack of autophagy in regard to proliferation and differentiation, and exhibit normal inflammatory responses.

It has been previously recognized that TNF induces IEC apoptosis in the context of IBD and in murine disease models. Our results show that autophagy-deficient IECs are much more sensitive to cytokine-induced apoptosis. Therefore we wondered whether TNF blockade would ameliorate the increased IEC death and intestinal pathology observed in colitic *Atg16l1*^VC^ mice. Indeed, TNF blockade significantly attenuates disease severity and reduces IEC apoptosis in *Atg16l1*^VC^ mice. These findings confirm that the exacerbated pathology in *Atg16l1*^VC^ mice is largely driven by TNF-induced IEC apoptosis (Figure 1).

**Figure 1.**
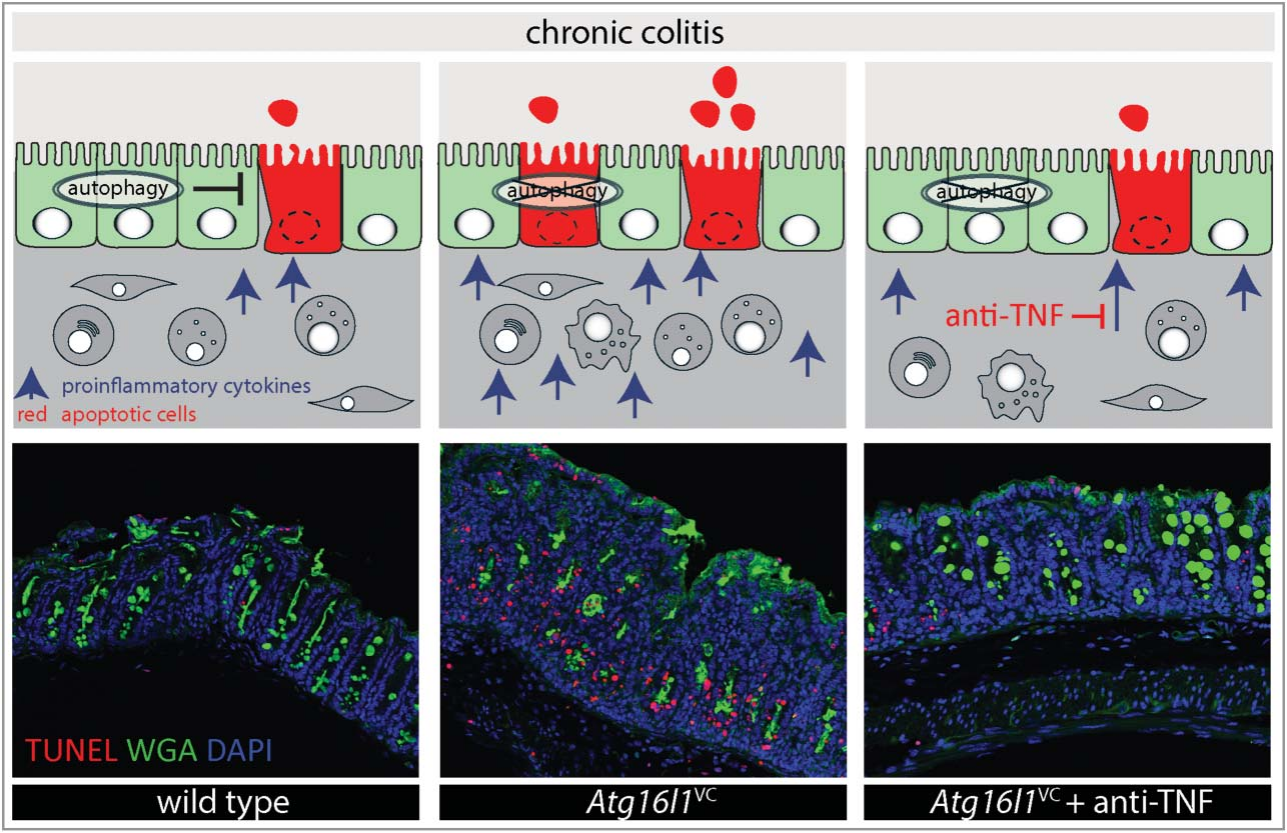
Autophagy limits cytokine-induced apoptosis of intestinal epithelial cells during chronic intestinal inflammation. Upper panels: Schematic representation of the protective role of autophagy in IECs during chronic intestinal inflammation. Lower panels: Representative immunofluorescence staining of apoptotic cells (TUNEL, red), counterstain (WGA, green) and nuclei (DAPI, blue) in colon sections taken from wild-type mice, *Atg16l1*^VC^ mice and *Atg16l1*^VC^ mice treated with a blocking antibody against TNF (anti-TNF), during experimental IBD. In wild-type IECs the apoptosis pathway is limited by autophagy, resulting in only a few apoptotic cells and moderate levels of inflammation (left panels). Selective ablation of autophagy in IECs leads to increased epithelial apoptosis, resulting in exacerbated inflammatory pathology (middle panels). These can be ameliorated by blocking TNF, the main driver of apoptosis (right panels). Lower panels reproduced with permission from Elsevier (color online).

TNF is a key cytokine in IBD pathology, and treatment strategies targeting TNF signaling are one of the most effective treatment regimens. Despite the proven importance of the cytokine, it is not completely understood how TNF mediates its pathogenic effects and thus how TNF blockade acts to promote disease resolution. Of note, there is considerable heterogeneity among IBD patients in terms of disease manifestations, inflammatory profile and treatment responsiveness. Indeed, although anti-TNF therapies can be very effective, approximately 40% of IBD patients do not respond to anti-TNF treatment and many more become refractory to treatment. TNF blockers are expensive therapeutics and, to date, it is not possible to predict whether a given patient will respond to anti-TNF therapies. Our results suggest that IBD patients harboring risk alleles that sensitize the epithelial barrier to apoptosis may be more likely to respond to anti-TNF therapies, and if confirmed, this could lead to more efficient deployment of such treatments.

